# Validation of Inertial Sensor-Based Step Detection Algorithms for Edge Device Deployment

**DOI:** 10.3390/s26030876

**Published:** 2026-01-29

**Authors:** Maksymilian Kisiel, Arslan Amjad, Agnieszka Szczęsna

**Affiliations:** Department of Computer Graphics, Vision and Digital Systems, Faculty of Automatic Control, Electronics and Computer Science, Silesian University of Technology, 44-100 Gliwice, Poland; makskisiel@interia.pl (M.K.); arslan.amjad.abbasi@polsl.pl (A.A.)

**Keywords:** step detection, inertial measurement unit (IMU), signal processing, gait analysis, wearable sensors, edge computing

## Abstract

Step detection based on measurements of inertial measurement units (IMUs) is fundamental for human activity recognition, indoor navigation, and health monitoring applications. This study validates and compares five fundamentally different step detection algorithms for potential implementation on edge devices. A dedicated measurement system based on the Raspberry Pi Pico 2W microcontroller with two IMU sensors (Waveshare Pico-10DOF-IMU and Adafruit ST-9-DOF-Combo) was designed. The implemented algorithms include Peak Detection, Zero-Crossing, Spectral Analysis, Adaptive Threshold, and SHOE (Step Heading Offset Estimator). Validation was performed across 84 measurement sessions covering seven test scenarios (Timed Up and Go test, natural and fast walking, jogging, and stair climbing) and four sensor mounting locations (thigh pocket, ankle, wrist, and upper arm). Results demonstrate that Peak Detection achieved the best overall performance, with an average F1-score of 0.82, while Spectral Analysis excelled in stair scenarios (F1 = 0.86–0.92). Surprisingly, upper arm mounting yielded the highest accuracy (F1 = 0.84), outperforming ankle placement. The TUG clinical test proved most challenging (average F1 = 0.68), while fast walking was easiest (F1 = 0.87). Additionally, a preliminary application to 668 clinical TUG recordings from the open-access FRAILPOL database revealed algorithm-specific failure modes when continuous gait assumptions are violated. These findings provide practical guidelines for algorithm selection in edge computing applications and activity monitoring systems.

## 1. Introduction

Step detection based on measurements of inertial sensors constitutes a fundamental problem in human activity recognition (HAR) with broad applications in indoor navigation, health monitoring, and fitness tracking [1,2]. The ability to accurately count steps and detect gait events forms the foundation for numerous applications ranging from consumer fitness devices to clinical assessment tools. As wearable technology becomes increasingly ubiquitous, the demand for reliable, computationally efficient step detection algorithms continue to grow.

The human gait cycle comprises two main phases: the stance phase, during which the foot maintains ground contact, and the swing phase, when the foot is airborne. Key events within this cycle—heel strike (HS) and toe-off (TO)—generate characteristic acceleration patterns that algorithms use for step counting [3]. Understanding these biomechanical patterns is essential for developing effective detection algorithms, as the signal characteristics vary significantly depending on walking speed, surface type, and individual gait patterns.

The clinical significance of accurate step detection extends beyond simple counting. In Parkinson’s disease monitoring, step length and cadence correlate strongly with disease progression, enabling objective tracking of motor symptoms [4]. Post-stroke rehabilitation programs utilize step counting to monitor patient recovery and adjust therapy intensity. In pedestrian dead reckoning (PDR) systems where GPS signals are unavailable, precise step counting combined with step length and heading estimation enables trajectory reconstruction for indoor navigation [2]. Consumer wearable devices rely on step detection for daily activity quantification, though accuracy varies significantly across devices and conditions [5,6].

The evolution of step detection methods spans from simple threshold-based approaches of the 2000s, through advanced wavelet transforms and ZUPT (Zero Velocity Update) methods in the 2010s, to contemporary deep learning solutions [7,8]. Despite this progress, achieving universal accuracy across real-world conditions remains challenging. Walking speed significantly affects signal characteristics—very slow walking (below $2\;\frac{\mathrm{km}}{h}$) generates signals difficult to distinguish from noise due to low amplitudes, while running (above $9\;\frac{\mathrm{km}}{h}$) produces high amplitudes with short stance phases [6]. Sensor placement introduces additional complexity, with accuracy differences between ankle, hip, and wrist mounting reaching 10–30 percentage points [9,10].

A critical emerging application domain is edge computing, where step detection must execute locally on resource-constrained microcontrollers rather than in the cloud [11]. This paradigm shift demands algorithms that balance accuracy with computational efficiency, real-time processing capability, and a minimal memory footprint. Edge deployment offers advantages including reduced latency, improved privacy, and operation without network connectivity—essential features for many healthcare and navigation applications.

However, existing literature provides fragmented results obtained under varying experimental conditions, making objective algorithm comparison difficult [12]. Studies typically evaluate single algorithms or limited scenarios, use different sensor configurations and sampling rates, and report inconsistent metrics. This fragmentation hinders practitioners from selecting appropriate algorithms for specific applications.

This study addresses these gaps by implementing and validating five fundamentally different step detection approaches within a unified experimental framework. The primary objectives are: (1) to design a low-cost, prototype measurement platform suitable for algorithm benchmarking; (2) to implement representative algorithms spanning threshold-based, spectral, adaptive, and hybrid approaches; and (3) to conduct comprehensive validation across diverse scenarios and sensor placements relevant to practical applications. Additionally, this work contributes to validation using the clinical Timed Up and Go (TUG) test recordings from FRAILPOL repository [13], bridging the gap between laboratory and clinical settings.

## 2. Materials and Methods

### 2.1. Hardware Platform

The prototype measurement system was designed to verify implementation of step detection algorithms for future edge deployment. The platform was based on the Raspberry Pi Pico 2W microcontroller featuring dual ARM Cortex-M33 cores operating at 150 MHz with 520 kB SRAM, 4 MB flash memory, and integrated Wi-Fi connectivity [14]. This microcontroller represents a typical target for edge computing applications, offering sufficient computational resources for real-time signal processing while maintaining low power consumption. The upgraded memory capacity (520 kB vs. 264 kB in the original Pico W) proved essential for reliable operation when combining the sensor acquisition with wireless data transmission.

Two IMU sensors with different characteristics were employed to assess algorithm robustness across hardware variations:

- Waveshare Pico-10DOF-IMU incorporating the InvenSense MPU-9250 nine-axis motion tracking device. This sensor provides a 3-axis accelerometer with a selectable full-scale range up to ±16 g, a 3-axis gyroscope with range up to ±2000°/s, and a 3-axis magnetometer.
- Adafruit ST 9-DoF Combo featuring the STMicroelectronics LSM6DSOX inertial module (accelerometer ±16 g, gyroscope ±2000°/s) combined with the LIS3MDL magnetometer. The LSM6DSOX includes an embedded machine learning core, though this feature was not utilized to ensure fair comparison.

Both sensors communicated via I^2^C bus. The sampling rate was configured to 100 Hz, providing sufficient temporal resolution for the gait event detection while remaining within typical ranges for clinical applications [15]. According to the Nyquist–Shannon theorem, this sampling rate adequately captures gait frequencies (typically 0.5–3 Hz) including higher harmonics. Data transmission utilized the UDP protocol over Wi-Fi to a host PC running the analysis software, achieving reliable transmission with minimal latency (<10 ms). The total hardware cost was approximately 50 USD (at prices from January 2026), which, while affordable for well-resourced institutions, may still present barriers in low- and middle-income countries where gait assessment tools are critically needed for conditions like stroke rehabilitation and cerebral palsy. This can also be part of an assessment for recognition and better care of people living with dementia [16,17,18]. Cost reduction to approximately 25–30 USD is achievable by eliminating the secondary IMU sensor and OLED display, retaining only the essential Raspberry Pi Pico 2W and single IMU configuration. Further cost optimization through alternative microcontroller platforms (e.g., ESP32-based solutions at approximately 5–10 USD) represents a promising direction for truly accessible gait monitoring tools. Bluetooth can also be selected for data transmission.

**Figure 1:**
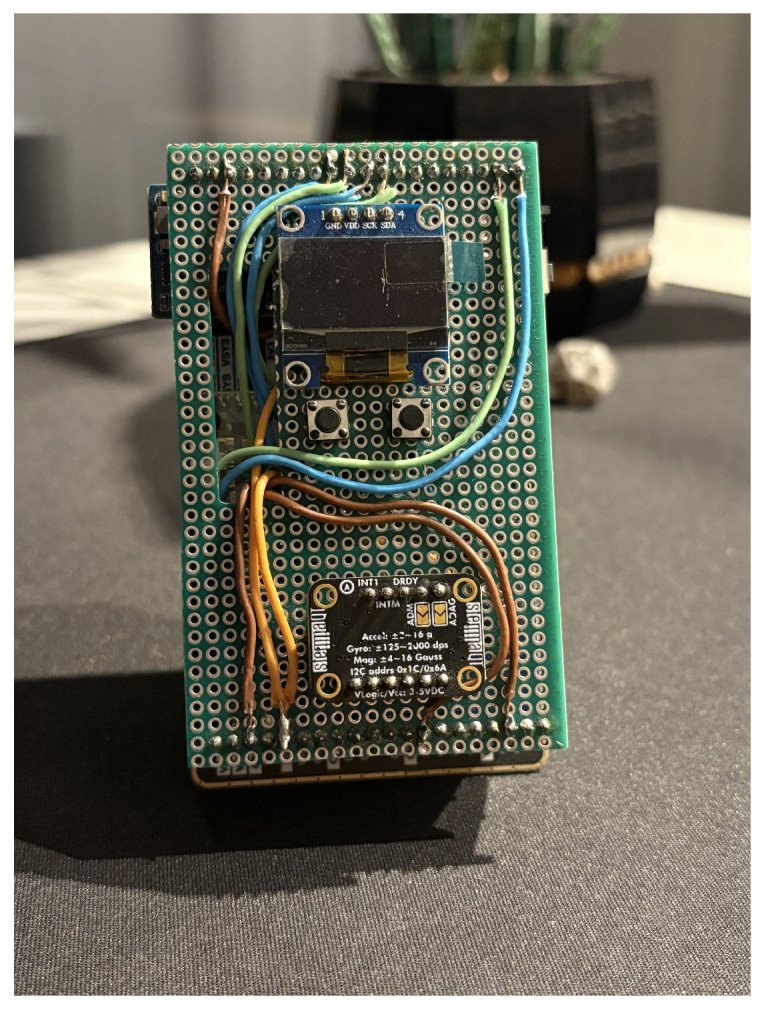
The assembled measurement platform showing the front panel with OLED display (top), tactile navigation buttons, and Adafruit ST 9-DoF IMU sensor (bottom). The Waveshare Pico-10DOF-IMU and Raspberry Pi Pico 2W microcontroller are mounted on the reverse side.

**Figure 2:**
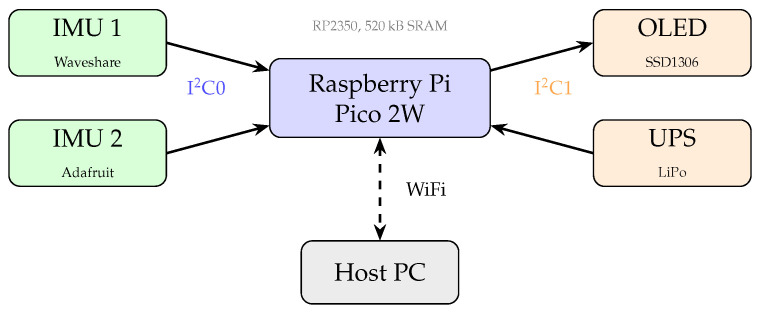
System architecture of the measurement platform. IMU sensors and UPS communicate via I^2^C0 bus (GP6/GP7), while the OLED display uses I^2^C1 (GP8/GP9). Sensor data is transmitted wirelessly to the host PC for offline analysis.

Figure 1 presents the assembled measurement platform, and Figure 2 presents the system architecture. The prototype integrates all components on a perforated PCB, with the Raspberry Pi Pico 2W mounted on the reverse side. The compact form factor (approximately 8×5 cm) enables comfortable body attachment using elastic bands.

### 2.2. Implemented Algorithms

Five step detection algorithms representing major methodological approaches were implemented in Python version 3.13 using the NumPy and SciPy libraries. All algorithms share common preprocessing but differ fundamentally in their detection strategies.

#### 2.2.1. Peak Detection

This method identifies local maxima in the filtered acceleration magnitude signal as potential steps. The algorithm operates on the assumption that each step produces a characteristic peak in acceleration corresponding to the heel strike event.

The implementation first computes the acceleration magnitude from the three-axis data and applies a Savitzky–Golay filter for smoothing. The signal is then centered by subtracting the median value (gravity baseline). An adaptive threshold is computed as the product of a 2 s rolling standard deviation and a configurable threshold parameter. Step candidates are identified using the SciPy find_peaks function with constraints on minimum height (adaptive threshold), minimum distance between peaks (default 0.3 s), and prominence (0.2). The minimum distance constraint limits detection to physiologically plausible cadences below 200 steps/min [19].

#### 2.2.2. Zero-Crossing

This approach detects moments when the DC-removed acceleration signal transitions through zero, corresponding to transitions between stance and swing phases. The acceleration magnitude is filtered and centered by subtracting the median (gravity reference).

To prevent multiple detections from signal oscillations around zero, the algorithm implements a hysteresis mechanism. A crossing is registered only when the signal rises above a positive hysteresis band and subsequently falls below a negative band of equal magnitude. Additionally, an inhibition period after each detection and minimum inter-step intervals (default 0.3 s) are enforced to ensure physiologically plausible step timing.

#### 2.2.3. Spectral Analysis

Operating in the frequency domain, this algorithm identifies the dominant gait frequency using Short-Time Fourier Transform (STFT) analysis. The acceleration magnitude signal is analyzed using overlapping windows (default 5 s with 50% overlap) with a Hanning window function to reduce spectral leakage.

For each time window, the power spectrum is computed, and the dominant frequency is identified within the physiological gait range (default 1.0–2.5 Hz). The median of dominant frequencies across all windows represents the estimated cadence. The total step count is calculated by multiplying this median frequency by the signal duration, and detected step times are distributed uniformly across the recording. This method excels with regular, periodic gait patterns but may struggle with variable cadence or activities involving frequent stops [20].

#### 2.2.4. Adaptive Threshold

This algorithm identifies steps through a two-phase process involving initial peak detection followed by amplitude-based filtering. First, all local maxima in the filtered acceleration signal are identified regardless of amplitude. For each candidate peak, the algorithm computes its amplitude as the difference between the peak value and the local minimum within a surrounding window (approximately 1 s).

The adaptive threshold is calculated as the mean of these amplitudes multiplied by a sensitivity parameter (default 0.6). Only peaks with amplitudes exceeding this threshold are retained as valid steps. This approach enables adaptation to varying signal strengths across different walking speeds and sensor placements without requiring manual threshold calibration [19].

#### 2.2.5. SHOE (Step Heading Offset Estimator)

The SHOE detector combines accelerometer and gyroscope data to identify stance phases when the foot is relatively stationary. Both acceleration and gyroscope magnitude signals are filtered using Savitzky–Golay smoothing and then normalized to the range [0, 1] using min–max scaling.

A combined signal is computed as a weighted sum: 70% normalized acceleration and 30% normalized gyroscope magnitude. Stance phases are detected using analyzing sliding windows (approximately 0.2 s) where the acceleration variance falls below a threshold and the mean absolute gyroscope reading is low. Step events are registered at these detected stance phases, with minimum inter-step spacing enforced.

If no stance phases are detected (common for non-foot mounting locations), the algorithm falls back to peak detection on the combined signal. Originally developed for foot-mounted sensors in navigation systems [21,22], this study evaluates its applicability to alternative mounting locations where the stationary assumption may not hold.

#### 2.2.6. Algorithm Parameters

Table 1 summarizes the key parameters used for each algorithm. Parameters were tuned empirically to achieve robust performance across diverse scenarios and mounting locations, following the principle of universal configuration without scenario-specific optimization.

The tolerance window for ground truth matching was set to ±0.3 s across all algorithms, accounting for human reaction time variability during manual annotation [15].

### 2.3. Signal Preprocessing

All algorithms shared common preprocessing steps to ensure fair comparison:

1. Acceleration magnitude computation: The three-axis acceleration vector is converted to a scalar magnitude (Euclidean norm), providing orientation-independent measurement.
2. Gravity compensation: The median acceleration value is subtracted from the signal to isolate dynamic acceleration components. The median is preferred over the mean, as it is more robust to transient high-amplitude events.
3. Low-pass filtering: A Savitzky–Golay filter removes high-frequency noise while preserving the temporal characteristics of gait events. This filter was chosen over conventional low-pass filters due to its superior preservation of peak amplitudes and locations.

### 2.4. Experimental Protocol

#### 2.4.1. Test Scenarios

Seven scenarios were designed to evaluate algorithm performance across diverse conditions representative of daily activities:

1. Timed Up and Go (TUG): A standardized clinical test involving rising from a chair, walking 3 m, turning around a marker, and returning to a seated position. This test incorporates multiple activity transitions (sitting–standing–walking–turning–walking–sitting) and is widely used for assessing mobility in elderly and neurological populations [4]. Each trial lasted approximately 10–15 s.
2. Natural walking: Level walking at self-selected comfortable pace (approximately $4–5\;\frac{\mathrm{km}}{h}$) along a 10 m corridor with turns at each end, repeated for 60 s. This represents the most common daily activity and serves as a baseline performance reference.
3. Fast walking: Increased pace (approximately $5–7\;\frac{\mathrm{km}}{h}$), testing algorithm performance at higher cadences. This scenario evaluates whether algorithms can adapt to shorter inter-step intervals.
4. Jogging: Light running (approximately $7–9\;\frac{\mathrm{km}}{h}$) with fundamentally different gait dynamics characterized by the absence of the double support phase and increased vertical acceleration amplitudes. This scenario tests algorithm robustness to gait pattern changes.
5. Stairs ascending: Climbing 28 wooden steps (rise: 18 cm, tread: 25 cm) at a natural pace. Stair climbing produces distinct acceleration patterns with asymmetric profiles compared to level walking.
6. Stairs descending: Descending 28 steps with typically higher impact amplitudes due to gravitational assistance. This scenario is particularly relevant for fall risk assessment applications.
7. Stairs combined: Sequential ascending and descending with direction change at the bottom, testing algorithm behavior during transitions between climbing modes.

#### 2.4.2. Sensor Mounting Locations

Four body locations representing common wearable device placements were evaluated:

- Front trouser pocket (thigh): Representing smartphone placement, the most convenient but potentially variable location due to loose coupling with body movement.
- Ankle of dominant leg: Traditional location for research-grade gait analysis providing direct measurement of the leg movement.
- Wrist of dominant hand: Standard smartwatch location, subject to arm swing artifacts unrelated to stepping.
- Upper arm: Alternative wearable location potentially offering more stable signals than wrist due to reduced degrees of freedom.

Sensors were secured using elastic fabric bands (buff-style), maintaining consistent orientation across trials. The sensor orientation was kept approximately constant with the Z-axis of the IMUs pointing outward from the body surface.

#### 2.4.3. Data Collection

For each scenario–location combination, three trials were recorded, yielding 84 measurement sessions (7×4×3). A single healthy adult male participant (age: 24, height: 170 cm, weight: 63 kg) performed all trials to eliminate inter-subject variability in this initial validation study.

Ground truth was established through manual step annotation during recording. The participant served as their own annotator, pressing a wireless mouse button held in the dominant hand at each perceived heel strike while performing the walking task. This self-annotation approach was chosen to eliminate the need for an external observer and to leverage the participant’s proprioceptive awareness of their own gait events. For the 10 m corridor walks, the participant maintained awareness of their stepping rhythm without requiring visual confirmation. For stair scenarios, the tactile feedback from each step provided reliable timing cues. While this approach introduces human reaction time delays (typically 100–200 ms), it provides timestamps synchronized with the sensor data stream. A tolerance window of ±0.3 s was applied when matching detected steps to references, accounting for reaction time variability [15]. This tolerance window (approximately 1.5× the typical reaction time) ensures that systematic delays in button pressing do not penalize correctly timed detections.

Between trials, a 30 s rest period allowed for data verification and preparation. All data were stored in CSV format containing timestamps, nine-axis IMU readings from both sensors, and manually annotated step markers.

### 2.5. Validation with FRAILPOL Repository

Algorithm validation was extended using clinical TUG test recordings [13]. These data were collected from patients in clinical settings using different IMU hardware, complementing the controlled-environment measurements. An interpreter module was developed to convert external data formats (varying column structures, sampling rates, and coordinate systems) to the unified analysis format used in this study. The clinical dataset will be published as a public repository to facilitate reproducible research.

### 2.6. Evaluation Metrics

Algorithm performance was quantified using standard classification metrics:

- Precision (P): $\frac{TP}{TP+FP}$—proportion of detected steps that were correct, penalizing false positives.
- Recall (R): $\frac{TP}{TP+FN}$—proportion of actual steps that were detected, penalizing missed steps.
- F1-score: $2\cdot \frac{P\cdot R}{P+R}$—harmonic mean, providing balanced assessment of precision and recall.

where TP is true positives (correctly detected steps), FP is false positives (spurious detections), and FN is false negatives (missed steps).

Additionally, execution time was measured using a standard PC (AMD Ryzen 7, 32 GB RAM) to assess computational feasibility for edge deployment. While not directly comparable to microcontroller performance, relative execution times indicate algorithmic complexity.

## 3. Results

### 3.1. Overall Algorithm Performance

Table 2 summarizes the mean F1-scores across all scenarios and mounting locations. Peak Detection achieved the highest overall performance (F1 = 0.82), followed by Spectral Analysis (0.81), Adaptive Threshold (0.77), Zero-Crossing (0.76), and SHOE (0.75). The performance range indicates substantial variability across experimental conditions, with all algorithms achieving both excellent (>0.90) and poor (<0.60) results depending on the specific scenario–location combination.

Notably, Zero-Crossing exhibited the highest variability (standard deviation 0.18), indicating strong sensitivity to experimental conditions. Spectral Analysis showed the most consistent performance (standard deviation 0.11), though its minimum F1-score (0.61) was higher than other algorithms, suggesting a floor effect in challenging conditions.

### 3.2. Impact of Test Scenario

Table 3 presents F1-scores stratified by test scenario. Fast walking yielded the highest average accuracy (0.87), benefiting from strong, regular signals. The TUG test proved most challenging (mean F1 = 0.68), with all algorithms except Peak Detection falling below 0.80. Stair scenarios achieved above-average performance (0.78–0.85), with Spectral Analysis dominating these conditions.

The Spectral Analysis algorithm achieved its best performance in the stairs combined scenario (F1 = 0.92), the highest single-scenario result in the study. Conversely, Zero-Crossing achieved its worst performance in the TUG scenario (F1 = 0.56), representing near-random detection performance. Jogging presented challenges for frequency-domain methods (Spectral: 0.71) and fusion approaches (SHOE: 0.69), likely due to the fundamentally different gait dynamics compared to walking.

### 3.3. Impact of Sensor Mounting Location

Table 4 reveals unexpected findings regarding optimal sensor placement. Upper arm mounting achieved the highest mean F1-score (0.84), surpassing traditional ankle placement (0.80). Wrist mounting performed worst overall (0.73), likely due to arm swing artifacts unrelated to stepping.

The thigh pocket location, representing typical smartphone placement, achieved the second-highest mean performance (0.81), with Peak Detection reaching 0.89—the highest location-specific result for this algorithm. Interestingly, Zero-Crossing performed best at the wrist (F1 = 0.84), contrasting sharply with its poor ankle performance (0.71). This algorithm–location interaction suggests that optimal algorithm selection depends on the intended device form factor.

### 3.4. Detailed Scenario–Location Analysis

Table 5 presents complete results for all scenario–location combinations, enabling identification of the best- and worst-performing configurations.

The highest individual F1-scores (0.98) were achieved by Peak Detection for thigh-mounted fast walking and stairs combined scenarios. The lowest score (0.33) was recorded by Zero-Crossing at the ankle during TUG tests, indicating near-complete algorithm failure in this configuration.

### 3.5. Sensor Comparison

Both sensors (Waveshare MPU9250 and Adafruit LSM6DSOX) exhibited comparable detection accuracy, with F1-score differences typically below 5 percentage points for identical experimental conditions. After uniform preprocessing, hardware-specific characteristics were largely normalized, suggesting that algorithm selection matters more than sensor choice within the tested quality range.

### 3.6. Preliminary Application to Clinical TUG Data

To evaluate algorithm generalization beyond controlled experiments, the implemented detection methods were applied to an independent clinical dataset comprising 668 TUG test recordings from the FRAILPOL repository [13]. Each recording included five synchronized IMU sensors (left/right ankle, left/right wrist, sacrum), yielding 3340 individual analyses. Notably, no ground truth step counts were available for this dataset, precluding F1-score computation. Instead, we analyzed detection plausibility based on expected TUG step counts and inter-algorithm consistency.

The utility of analyzing clinical data without ground truth lies in identifying algorithm failure modes that may not manifest in controlled laboratory conditions. Clinical populations exhibit greater variability in gait patterns, including asymmetric stepping (e.g., post-stroke hemiparesis), shuffling gait (Parkinson’s disease), and assistive device use. Furthermore, clinical IMU mounting may be less precise than research protocols, introducing additional signal variability. By examining whether algorithms produce physiologically plausible outputs across diverse clinical recordings, we can assess their robustness to real-world deployment conditions. Algorithms that frequently produce implausible results (e.g., zero steps or hundreds of steps for a 20 s TUG) are unlikely to perform reliably in clinical practice, regardless of their accuracy in controlled settings.

Table 6 summarizes the detection results. The proportion of recordings yielding plausible step counts (4–20 steps) varied dramatically across algorithms, revealing fundamental limitations when transitioning from controlled to clinical conditions. The plausible range was defined conservatively: the lower bound (4 steps) identifies complete algorithm failures producing near-zero detections, while the upper bound (20 steps) accommodates shorter stride lengths typical in the elderly and mobility-impaired populations walking the 6 m TUG distance (3 m each way around a marker). This range primarily serves to identify gross algorithmic errors (e.g., detecting hundreds or thousands of steps) rather than to establish precise clinical norms.

#### 3.6.1. Algorithm-Specific Failure Modes

The SHOE algorithm exhibited bimodal failure on clinical data, with 41.5% of recordings in the plausible range. The algorithm detected either very few steps (1–3) or implausibly many (hundreds to thousands). This behavior stems from its reliance on detecting “stance phases” where the sensor experiences near-zero velocity—an assumption valid only for foot-mounted sensors. At wrist, arm, and sacrum locations, true stationary periods rarely occur during walking. When the stance detection threshold is not met, the algorithm’s fallback mechanism produces minimal detections; conversely, noisy signals matching the threshold criteria generate excessive false positives.

Spectral Analysis systematically overestimated step counts (median 18, P90 = 51), achieving only 60.5% plausible results. The algorithm assumes a continuous periodic gait throughout the recording, computing total steps as the product of the dominant frequency and the recording duration. However, TUG tests inherently include non-walking segments (sitting, standing up, turning, sitting down), causing the algorithm to “count steps” during stationary periods. For a 20 s recording with an estimated cadence of 1.1 Hz, this yields approximately 22 steps, regardless of actual walking duration.

Peak Detection, Adaptive Threshold, and Zero-Crossing demonstrated robust performance (86.5–93.7% plausible), with Zero-Crossing achieving the highest consistency. These algorithms operate on instantaneous signal characteristics rather than global assumptions, making them inherently more suitable for variable-activity recordings. The 99th percentile values (26–182) indicate occasional extreme failures, likely attributable to sensor artifacts, patient-specific gait abnormalities, or recording errors rather than systematic algorithm limitations.

#### 3.6.2. Bilateral Sensor Consistency

Comparison between left and right sensors provided an indirect validation metric. For recordings where both sensors were available, the mean absolute difference between bilateral ankle sensors was 3.0 steps (SD = 9.7), while bilateral wrist sensors showed better agreement at 2.5 steps (SD = 4.0). The higher ankle variability may reflect asymmetric gait patterns common in clinical populations (e.g., post-stroke hemiparesis, joint replacements).

#### 3.6.3. Comparison to Step Detection Utilizing Event Detection Based on Stride Segmentation

For more reliable verification, the results were compared to those obtained by a recognized gait analysis library. This library relies on precisely calibrated IMU signals from two feet. It should be noted that the algorithms (Peak Det., Zero Cross., Spectral, Adaptive, and SHOE) used did not require a calibration phase, meaning knowledge of the sensor orientation during the measurements.

**Figure 3:**
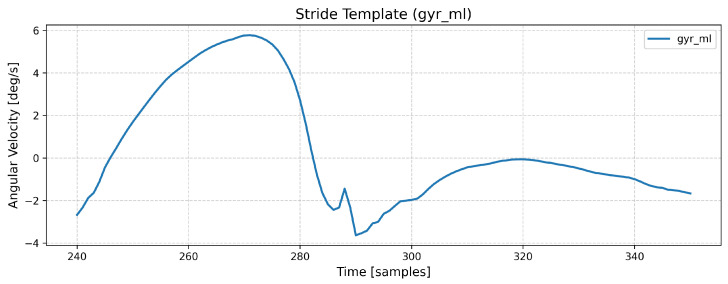
The stride template signal.

Step detection was performed using both ankle-worn sensors. The raw IMU signals from these sensors were processed using a Python library, Gaitmap (v.2.3.0) [12]. First, the raw IMU signals recorded from both sensors were processed. The processing involves sensor-specific coordinate transformations. The signals were transformed from the sensor frame to a foot-based coordinate system (ML (medial to lateral), PA (posterior to anterior), and SI (superior to inferior)). Afterwards, the library segmented the signals based on strides using a Dynamic Time Warping (DTW) approach [23]. The DTW method recognized stride boundaries by determining the best alignments between the pre-defined template (as in Figure 3) and the IMU signal. The stride template was derived from healthy older adult gait patterns. The function returns a list of detected strides for each foot with start and end indices.

Afterwards, the lists of strides from each participant were used for gait event detection. Gait events (i.e., Initial Contact [IC] and Terminal Contact [TC]) were identified using the Herzer event detection algorithm [24,25]. The algorithm was configured with parameters optimized for older adult gait patterns (min_vel_search_win_size_ms = 130, mid_swing_peak_prominence = 0.03). Finally, the step count was calculated as the total number of IC events across both limbs, with each IC representing one step.

**Figure 4:**
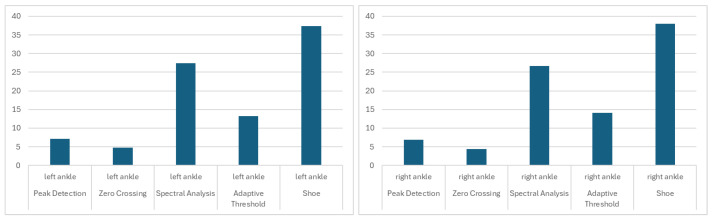
The mean differences between the stride count for left/right ankle IMU signals, based on selected algorithms and the Gaitmap implementation of the Herzer event detection algorithm.

**Figure 5:**
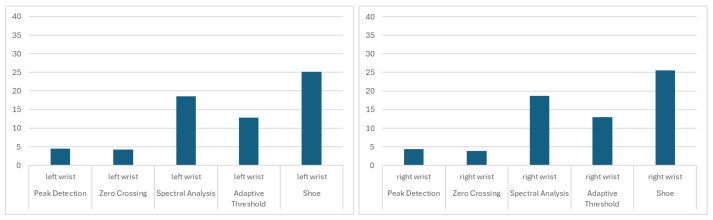
The mean differences between the stride count for left/right wrist IMU signals, based on selected algorithms and the Gaitmap implementation of the Herzer event detection algorithm.

**Figure 6:**
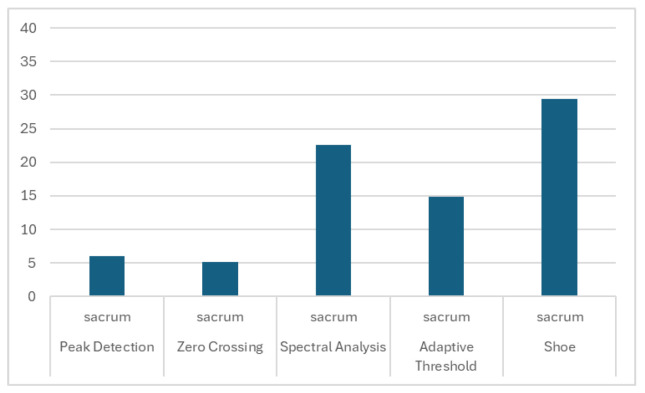
The mean differences between the stride count for sacrum back IMU signals, based on selected algorithms and the Gaitmap implementation of the Herzer event detection algorithm.

The smallest difference below the 5-step average was for the Zero Crossing algorithm for the right wrist (3.90 ± 2.79). It was also low for the left wrist (4.24 ± 3.07). Acceptable results were also achieved for the left ankle (4.77 ± 4.21) and right ankle (4.41 ± 4.04). The Peak Detection algorithm achieved acceptable results for signals from the right (4.42 ± 4.79) and left (4.47 ± 4.56) wrists. The results are presented in Figure 4, Figure 5 and Figure 6.

#### 3.6.4. Implications for Clinical Deployment

These findings suggest that algorithm selection is critical for clinical applications. For TUG-style assessments involving activity transitions:

- Strong recommendation: Zero-Crossing, which obtains results that are acceptable and close to those based on an advanced step analysis method requiring specific assembly and accurate calibration of coordinate systems.
- Recommended: Peak Detection and Adaptive Threshold algorithms, which do not assume continuous walking.
- Not recommended: SHOE (designed for foot-mounted navigation sensors) and Spectral Analysis (assumes stationary gait frequency) without additional activity segmentation preprocessing.

Future clinical implementations should incorporate activity recognition to segment walking periods before applying step detection, or they should employ algorithms specifically designed for intermittent gait patterns.

## 4. Discussion

### 4.1. Algorithm Selection Guidelines

The results support scenario-specific algorithm recommendations for practitioners and align with findings from commercial device implementations.

Peak Detection offers the best overall balance of accuracy (F1 = 0.82) and robustness across conditions, making it suitable as a general-purpose solution. Its computational efficiency (2.5 ms per 60 s recording on PC) and conceptual simplicity supports straightforward edge implementation. The algorithm’s adaptive threshold mechanism enables automatic adjustment to varying signal amplitudes without manual calibration—a design principle shared with the Bosch BMA421 embedded step counter, achieving 94% accuracy in commercial smartwatches [26,27].

Spectral Analysis demonstrated exceptional performance for stair navigation (F1 = 0.86–0.92)—a critical activity for home-based elderly monitoring where stair climbing ability indicates functional capacity. Its fastest execution time (1.2 ms) further supports edge deployment, though the 8 s analysis window requires larger memory buffers compared to streaming algorithms. The frequency-domain approach excels when gait is regular and periodic, making it ideal for steady-state activities. However, performance degraded for irregular patterns (TUG: 0.66, jogging: 0.71), limiting applicability to transition-heavy scenarios.

Zero-Crossing achieved top performance for fast walking (F1 = 0.89) but exhibited catastrophic failure in TUG scenarios (F1 = 0.56), indicating poor generalization to complex activity sequences. The algorithm’s reliance on consistent signal polarity changes makes it vulnerable to activities where acceleration patterns deviate from sinusoidal waveforms. Surprisingly, Zero-Crossing performed best at the wrist location, suggesting potential for smartwatch applications, when combined with activity recognition to filter unsuitable contexts. Notably, this poor TUG performance in controlled single-subject experiments contrasts with the algorithm’s strong results (93.7% plausible) on the clinical FRAILPOL dataset. This discrepancy likely reflects differences in sensor hardware, mounting precision, and the absence of ground truth in clinical data—high plausibility rates indicate consistent detection within expected ranges but do not guarantee accuracy against true step counts.

Adaptive Threshold provided reliable performance across most conditions (mean F1 = 0.77), with particular strength at the ankle location (F1 = 0.87). Its continuous threshold adjustment mechanism—similar to the dynamic thresholding used in the Analog Devices ADXL367 pedometer implementation [28]—offers robustness to gradual changes in walking speed but may struggle with the abrupt transitions characteristic of the TUG test.

The SHOE algorithm, despite theoretical advantages from gyroscope fusion, underperformed expectations (mean F1 = 0.75). This likely reflects its original design for foot-mounted sensors in navigation applications, where the “zero velocity” assumption during stance phases is most valid [22]. At alternative mounting locations (wrist, arm, thigh), the sensor rarely experiences true stationary periods during walking, violating the algorithm’s fundamental assumption.

### 4.2. Mounting Location Considerations

The finding that upper arm placement (F1 = 0.84) outperformed ankle mounting (F1 = 0.80) contradicts common assumptions favoring leg-mounted sensors [9]. Several mechanisms may explain this result:

1. Signal regularity: The arm exhibits pendulum-like motion during walking with consistent frequency matching the step cadence. Unlike the leg, which experiences complex acceleration patterns during stance and swing phases, arm motion approximates a simple harmonic oscillator.
2. Reduced impact artifacts: Ankle sensors experience high-amplitude impacts at heel strike that can saturate sensors or introduce ringing artifacts. The arm, being mechanically isolated from ground contact, produces cleaner signals.
3. Bilateral coupling: Both arms move in coordinated opposition with the legs, effectively averaging left and right step signals rather than detecting only one leg’s motion.

**Figure 7:**
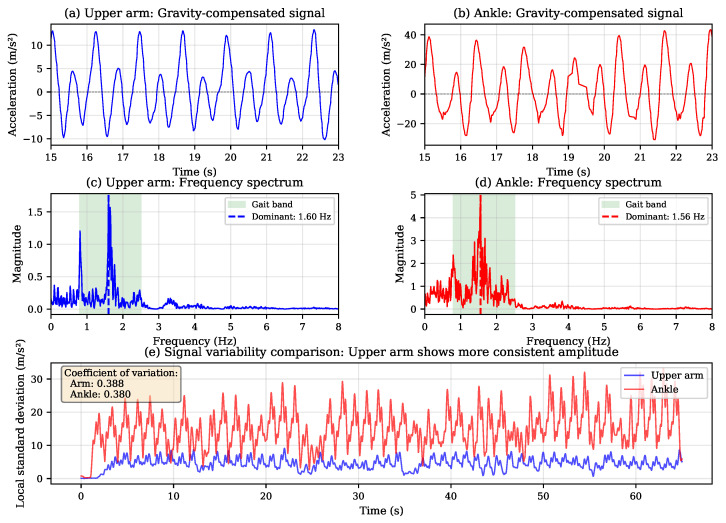
Signal comparison between upper arm and ankle sensor mounting during normal walking. (**a**,**b**) Time-domain signals after gravity compensation showing distinct waveform morphologies. (**c**,**d**) Frequency spectra with dominant gait frequencies marked. (**e**) Local signal variability comparison demonstrating amplitude consistency.

Figure 7 illustrates the signal characteristics at upper arm versus ankle mounting locations during normal walking. The time-domain comparison (panels a, b) reveals that arm signals exhibit smoother, more sinusoidal waveforms compared to the ankle’s sharper impact transients at heel strike. The frequency analysis (panels c, d) shows similar dominant frequencies in both signals (approximately 1.6 Hz), confirming that arm motion is well coupled to stepping cadence. Panel (e) presents the local signal variability over time, quantified as rolling standard deviation. While both locations show comparable coefficients of variation in this example, the arm signal’s more regular waveform morphology facilitates more consistent peak detection across varied conditions.

Wrist placement, despite its convenience for smartwatch applications, yielded the lowest overall accuracy (F1 = 0.73). This aligns with literature noting wrist-worn device limitations [5,6]. The wrist experiences additional rotational motion from hand gestures and object manipulation unrelated to stepping. Interestingly, Zero-Crossing performed best at the wrist (F1 = 0.84), suggesting algorithm-specific adaptation could improve commercial device accuracy.

The thigh pocket location (F1 = 0.81) representing smartphone placement performed well despite loose mechanical coupling. This supports the viability of smartphone-based step counting applications, though performance depends heavily on consistent phone orientation and secure pocket fit.

### 4.3. Clinical Test Challenges

The TUG test’s low detection rates (mean F1 = 0.68) highlight fundamental limitations of current approaches when gait transitions (sitting–standing–walking–turning) occur. This has significant implications for clinical applications where TUG is a standard mobility assessment tool [4].

Analysis of failure modes reveals that algorithms struggle with:

- Sit-to-stand transitions: Large vertical accelerations during rising mimic step signatures.
- Turning: Rotational motion produces complex acceleration patterns unlike straight walking.
- Variable cadence: Initial acceleration and final deceleration phases produce irregular step timing.

Future clinical applications require either activity-aware algorithm switching or the development of methods robust to transition artifacts. Multi-sensor fusion approaches combining accelerometer, gyroscope, and potentially pressure sensors may address these limitations.

### 4.4. Implications for Edge Computing

All implemented algorithms demonstrated execution times under 5 ms for 60 s recordings on standard PC hardware. While direct extrapolation to microcontroller performance is imprecise, the relative simplicity of operations (filtering, peak finding, FFT) suggests that real-time implementation is feasible on platforms like the Raspberry Pi Pico 2W.

Commercial step counting implementations in smartphones and smartwatches provide useful reference points for embedded design. Typical configurations employ 2–4 s analysis windows at 50–100 Hz sampling rates, resulting in buffer sizes of 100–400 samples per axis [26,28]. The Bosch BMA421 accelerometer, commonly used in smartwatches (e.g., PineTime), implements an embedded step counter, achieving 94% average accuracy with 25 µA current consumption [27]. The Analog Devices ADXL345/ADXL367 series utilizes 32–512 sample FIFO buffers with interrupt-driven processing at 50 Hz, achieving 97% accuracy for wrist-worn applications [28]. These implementations demonstrate that windowed peak detection algorithms operating on 4 s buffers (200 samples at 50 Hz, approximately 1.2 kB for 3-axis 16-bit data) can achieve commercial-grade accuracy.

Extrapolating to our implementation, the Raspberry Pi Pico 2W with its RP2350 microcontroller provides 520 kB SRAM and 4 MB flash memory [14]. Table 7 presents estimated memory requirements for each algorithm based on typical buffer configurations.

Memory requirements pose a more significant constraint for edge deployment than computation. Spectral Analysis requires buffering several seconds of data for FFT computation (8 s at 100 Hz = 4.8 kB for 3-axis data), while Peak Detection and Zero-Crossing can operate on streaming data with minimal memory. For severely memory-constrained devices, the latter algorithms offer advantages despite slightly lower accuracy.

The Raspberry Pi Pico 2W’s dual-core ARM Cortex-M33 architecture enables potential parallelization: one core handling sensor data acquisition via I^2^C while the second performs detection algorithms. This architecture mirrors common wearable device designs and supports the practical relevance of the tested platform. During initial development on the original Pico W (264 kB SRAM), memory constraints occasionally required system restarts due to heap exhaustion when running analysis algorithms alongside WiFi communication. The upgraded Pico 2W (520 kB SRAM) resolved these limitations, confirming that embedded step detection is feasible within the 50–100 kB typical working memory budget observed in commercial implementations.

#### Empirical Validation on Raspberry Pi Pico 2W

To validate the theoretical feasibility analysis, all five algorithms were implemented in MicroPython and tested directly on the Raspberry Pi Pico 2W hardware. Table 8 presents execution times and detection accuracy for an 8 s segment of normal walking data at the original sampling rate (≈100 Hz), comparing upper arm and ankle sensor placements.

The empirical results confirm that upper arm placement yields superior detection accuracy for event-based algorithms (Peak Detection, Zero-Crossing, Adaptive Threshold), while the SHOE algorithm performs better at the ankle location, as expected from its foot-mounted sensor design origins. Notably, Adaptive Threshold achieved perfect detection (F1 = 1.0) on the upper arm test segment.

Execution times of 400–650 ms for 8 s batch processing translate to approximately 50–80 ms of computation per second of sensor data. For real-time streaming applications with smaller analysis windows (e.g., 1–2 s), the per-window execution time would be proportionally reduced, enabling comfortable real-time operation with significant processing margin. The notably faster Spectral Analysis times (17–22 ms) reflect the simplified frequency estimation approach used in the memory-constrained MicroPython implementation, which employs zero-crossing rate estimation rather than full FFT computation.

These results demonstrate that the practical edge deployment of step detection algorithms on low-cost microcontrollers is not only theoretically feasible but empirically validated, with detection accuracy comparable to PC-based implementations.

### 4.5. Limitations and Future Work

Several limitations warrant acknowledgment:

- Single participant: All controlled experiments involved one healthy adult, limiting generalization to diverse populations (varying height, weight, age, gait pathologies). Gait dynamics vary substantially across demographic groups—elderly individuals typically exhibit reduced stride length and cadence, while pathological conditions such as Parkinson’s disease produce a characteristic shuffling gait with reduced arm swing amplitude. The algorithms validated here may require parameter re-tuning for such populations. However, the complementary analysis of 668 clinical TUG recordings from the FRAILPOL repository (Section 3.6) partially addresses this limitation by demonstrating algorithm behavior across diverse clinical populations, including elderly patients with varying mobility impairments. The finding that event-based algorithms (Peak Detection, Zero-Crossing) maintained 86–94% plausible detection rates on clinical data suggests reasonable generalization potential, though definitive validation with ground truth annotations remains necessary. Future studies should include broader participant cohorts with stratification by demographic and clinical characteristics.
- Controlled environment: Testing occurred in a home environment on level floors and standard stairs. Real-world conditions (uneven terrain, crowds, variable surfaces, outdoor environments) may introduce additional challenges.
- Manual annotation: Ground truth establishment incurred human reaction delays (≈100–200 ms). While the ±0.3 s tolerance window mitigated timing errors, systematic bias in annotation may affect absolute metrics. Video-based annotation or force plate validation would provide a more accurate reference.
- Fixed parameters: Universal algorithm parameters were prioritized over scenario-specific optimization. Adaptive parameter tuning or machine learning-based approaches may achieve higher accuracy at the cost of increased complexity.
- Clinical data without ground truth: The TUG dataset analysis was limited to plausibility assessment due to the absence of reference step counts. Future clinical validation should incorporate video-based annotation or instrumented walkways for definitive accuracy evaluation.

Future research directions include: (1) expanded participant cohorts with clinical populations; (2) machine learning algorithm integration for comparison with classical methods; (3) on-device implementation and power consumption characterization; and (4) validation in free-living conditions.

## 5. Conclusions

This study validated five step detection algorithms across 84 measurement sessions encompassing diverse scenarios and sensor placements. The unified experimental framework enables direct algorithm comparison, addressing fragmentation in existing literature. Key findings include:

1. Peak Detection provides robust general-purpose performance (F1 = 0.82 ± 0.12), with computational efficiency suitable for edge devices (2–3 ms execution time). Its adaptive threshold mechanism enables deployment without manual calibration, similar to commercial implementations in the Bosch BMA421 accelerometer.
2. Spectral Analysis excels for stair navigation (F1 = 0.86–0.92), making it particularly valuable for home-based activity monitoring of elderly populations, where stair climbing indicates functional capacity. The 8 s analysis window requires approximately 10 kB RAM, feasible on the Raspberry Pi Pico 2W (520 kB SRAM available).
3. Upper arm mounting unexpectedly outperformed ankle placement (F1 = 0.84 ± 0.04 vs. 0.80 ± 0.07), suggesting reconsideration of optimal sensor locations for wearable device design. The regular, pendulum-like arm motion during walking may produce more algorithm-friendly signals than complex leg dynamics.
4. Clinical transition scenarios (TUG) remain challenging (mean F1 = 0.68 ± 0.10), indicating the need for algorithms capable of handling activity transitions. This limitation has direct implications for clinical mobility assessment applications.
5. Algorithm–location matching matters: Zero-Crossing performed best at the wrist (F1 = 0.84 ± 0.06) despite an overall lower ranking, suggesting that customized algorithm selection based on device form factor can substantially improve accuracy.
6. Edge deployment is feasible: Memory requirements (1.5–10 kB, depending on the algorithm) and execution times (<5 ms) are compatible with commercial embedded implementations using 2–4 s analysis windows at 50–100 Hz sampling rates.
7. Clinical generalization requires algorithm awareness: Application to 668 clinical TUG recordings revealed that algorithms assuming a continuous gait (Spectral Analysis, SHOE) fails dramatically on variable-activity data, while event-based methods (Zero-Crossing, Peak Detection) maintain 86–94% plausible detection rates, even without ground truth validation.

The developed measurement platform (hardware cost ≈50 USD) and analysis software are publicly available, enabling reproducible research and algorithm benchmarking.

These results provide practical guidelines for edge computing applications and wearable device development. Algorithm selection should consider the target scenario, sensor placement, and computational constraints. For general-purpose applications, Peak Detection offers the best balance of accuracy and simplicity. For specific use cases (stair monitoring, fast walking detection), specialized algorithms may provide superior performance.

## Figures and Tables

**Table 1:** Algorithm parameters used in validation experiments. Window sizes are in seconds, thresholds are dimensionless multipliers, and frequency ranges are in Hz.

Algorithm	Parameter	Value	Description
Peak Detection	Window size	0.6 s	Smoothing window for Savitzky–Golay filter
	Threshold	0.5	Adaptive threshold multiplier (×rolling SD)
	Min. step interval	0.35 s	Minimum time between detected steps
Zero-Crossing	Window size	0.5 s	Signal smoothing window
	Min. step interval	0.4 s	Refractory period after detection
	Hysteresis band	0.3 m/s^2^	Threshold band to prevent oscillation
Spectral Analysis	Window size	8.0 s	STFT analysis window length
	Overlap	0.8	Window overlap ratio (80%)
	Freq. range	[0.8, 2.0] Hz	Valid gait frequency band
Adaptive Threshold	Window size	0.8 s	Local amplitude calculation window
	Sensitivity	0.5	Threshold sensitivity (× mean amplitude)
	Min. step interval	0.4 s	Minimum inter-step timing
SHOE	Window size	0.3 s	Stance phase detection window
	Threshold	9.0	Combined signal threshold
	Min. step interval	0.35 s	Minimum step spacing

**Table 2:** Mean F1-scores by algorithm across all experimental conditions. Values represent averages over 84 recordings (7 scenarios × 4 mounting locations × 3 trials, both sensors averaged).

Metric	Peak Det.	Zero Cross.	Spectral	Adaptive	SHOE
Mean F1-score	0.82	0.76	0.81	0.77	0.75
Standard deviation	0.12	0.18	0.11	0.14	0.12
Range (min–max)	0.51–0.98	0.33–0.96	0.61–0.97	0.50–0.95	0.50–0.93
Execution time (ms)	2.5	1.8	1.2	3.1	4.2

**Table 3:** Mean F1-scores (±standard deviation) by test scenario (averaged across mounting locations, n=12 per scenario). Bold indicates best-performing algorithm for each scenario.

Scenario	Peak Det.	Zero Cross.	Spectral	Adaptive	SHOE	Mean
TUG	**0.79** ± 0.13	0.56 ± 0.17	0.66 ± 0.04	0.77 ± 0.15	0.64 ± 0.09	0.68 ± 0.10
Natural walking	0.81 ± 0.15	0.77 ± 0.18	**0.86** ± 0.07	0.83 ± 0.15	0.80 ± 0.02	0.81 ± 0.04
Fast walking	0.87 ± 0.12	**0.89** ± 0.10	**0.89** ± 0.14	0.82 ± 0.11	0.86 ± 0.05	0.87 ± 0.03
Jogging	**0.84** ± 0.11	**0.84** ± 0.11	0.71 ± 0.11	0.69 ± 0.03	0.69 ± 0.12	0.75 ± 0.08
Stairs ascending	0.76 ± 0.16	0.79 ± 0.11	**0.88** ± 0.03	0.73 ± 0.19	0.72 ± 0.05	0.78 ± 0.07
Stairs descending	0.83 ± 0.15	0.80 ± 0.05	**0.86** ± 0.04	0.82 ± 0.15	0.77 ± 0.04	0.82 ± 0.04
Stairs combined	0.87 ± 0.09	0.84 ± 0.07	**0.92** ± 0.03	0.83 ± 0.04	0.81 ± 0.04	0.85 ± 0.04

**Table 4:** Mean F1-scores (±standard deviation) by mounting location (averaged across scenarios, n=21 per location). Bold indicates best-performing algorithm for each location.

Location	Peak Det.	Zero Cross.	Spectral	Adaptive	SHOE	Mean
Ankle	**0.87** ± 0.10	0.71 ± 0.19	0.83 ± 0.12	**0.87** ± 0.10	0.76 ± 0.05	0.80 ± 0.07
Upper arm	0.87 ± 0.05	0.82 ± 0.16	**0.89** ± 0.10	0.83 ± 0.05	0.78 ± 0.08	0.84 ± 0.04
Wrist	0.67 ± 0.13	**0.84** ± 0.06	0.74 ± 0.10	0.62 ± 0.09	0.76 ± 0.14	0.73 ± 0.08
Thigh pocket	**0.89** ± 0.08	0.76 ± 0.15	0.83 ± 0.12	0.82 ± 0.12	0.73 ± 0.10	0.81 ± 0.06

**Table 5:** Mean F1-scores for all scenario–location combinations (averaged over 3 trials, both sensors). Values ≥ 0.90 are highlighted in bold.

Location	Scenario	Peak Det.	Zero Cross.	Spectral	Adaptive	SHOE
Thigh	TUG	0.84	0.65	0.70	0.83	0.68
Thigh	Natural walking	**0.91**	0.49	0.82	**0.92**	0.78
Thigh	Fast walking	**0.98**	**0.96**	**0.97**	**0.92**	0.82
Thigh	Jogging	0.89	**0.90**	0.65	0.70	0.54
Thigh	Stairs ascending	0.75	0.73	**0.90**	0.61	0.67
Thigh	Stairs descending	0.89	0.75	0.86	**0.94**	0.78
Thigh	Stairs combined	**0.98**	0.83	**0.94**	0.85	0.81
Wrist	TUG	0.61	0.78	0.61	0.53	0.50
Wrist	Natural walking	0.58	0.84	0.77	0.59	0.79
Wrist	Fast walking	0.70	0.74	0.65	0.67	**0.93**
Wrist	Jogging	**0.91**	0.89	0.65	0.67	0.83
Wrist	Stairs ascending	0.51	**0.91**	0.85	0.50	0.69
Wrist	Stairs descending	0.60	0.83	0.80	0.59	0.71
Wrist	Stairs combined	0.76	0.89	0.89	0.76	0.86
Upper arm	TUG	0.79	0.47	0.70	0.79	0.65
Upper arm	Natural walking	0.88	0.86	**0.94**	0.88	0.82
Upper arm	Fast walking	**0.93**	**0.92**	**0.97**	0.85	**0.90**
Upper arm	Jogging	0.87	0.89	0.88	0.73	0.74
Upper arm	Stairs ascending	0.88	0.86	**0.92**	0.86	0.78
Upper arm	Stairs descending	0.87	0.86	0.89	0.83	0.76
Upper arm	Stairs combined	0.89	**0.90**	**0.94**	0.85	0.81
Ankle	TUG	**0.94**	0.33	0.65	**0.92**	0.72
Ankle	Natural walking	0.89	0.87	0.89	**0.91**	0.80
Ankle	Fast walking	0.88	**0.91**	**0.97**	0.86	0.79
Ankle	Jogging	0.67	0.67	0.66	0.67	0.66
Ankle	Stairs ascending	0.89	0.66	0.84	**0.95**	0.75
Ankle	Stairs descending	**0.96**	0.76	0.89	**0.94**	0.81
Ankle	Stairs combined	0.84	0.74	0.89	0.86	0.76

**Table 6:** Step detection results on clinical TUG dataset (669 recordings, 5 sensors each). Plausible range defined as 4–20 steps based on the standard TUG protocol expectations. The highest plausible value is marked in bold.

Algorithm	Median	P10	P90	P99	% Plausible
Zero-Crossing	11	6	17	26	**93.7**
Adaptive Threshold	13	8	21	182	88.8
Peak Detection	13	7	21	41	86.5
Spectral Analysis	18	10	51	177	60.5
SHOE	12	1	56	246	41.5

**Table 7:** Estimated memory requirements for edge deployment on Raspberry Pi Pico 2W (520 kB SRAM available). Buffer estimates assume 100 Hz sampling with 16-bit resolution.

Algorithm	Buffer Size	RAM Est.	Notes
Peak Detection	0.6 s (60 samples)	∼2 kB	Streaming capable, minimal buffering
Zero-Crossing	0.5 s (50 samples)	∼1.5 kB	Streaming capable, hysteresis state only
Spectral Analysis	8.0 s (800 samples)	∼10 kB	Requires FFT buffer + Hanning window
Adaptive Threshold	0.8 s (80 samples)	∼3 kB	Local amplitude history required
SHOE	0.3 s (30 samples)	∼2 kB	Dual-sensor (accel + gyro) buffers

**Table 8:** Step detection performance on Raspberry Pi Pico 2W hardware. Tests conducted on 8 s normal walking segments (761–800 samples) with ground truth steps. Execution times represent complete batch processing of the recorded segment. Values of F1-Score ≥ 0.80 are highlighted in bold.

Algorithm	Upper Arm		Ankle	
	F1-Score	Time (ms)	F1-Score	Time (ms)
Peak Detection	**0.818**	535	0.632	436
Zero-Crossing	**0.800**	450	0.667	393
Spectral Analysis	0.788	22	0.788	17
Adaptive Threshold	**1.000**	638	0.769	497
SHOE	0.571	644	**0.880**	557

## Data Availability

The measurement data and source code are available at: https://github.com/revalew/Master-Thesis/tree/master/step_detection/analysis/experiments (accessed on 1 December 2025).

## Abbreviations

The following abbreviations are used in this manuscript:

IMU	Inertial Measurement Unit
HAR	Human Activity Recognition
TUG	Timed Up and Go
PDR	Pedestrian Dead Reckoning
SD	Standard Deviation
SHOE	Step Heading Offset Estimator
ZUPT	Zero Velocity Update
HS	Heel Strike
TO	Toe-Off
FFT	Fast Fourier Transform

## References

[1] Godfrey A., Del Din S., Barry G., Mathers J.C., Rochester L. (2015). Instrumenting Gait with an Accelerometer: A System and Algorithm Examination. Med. Eng. Phys..

[2] Kang W., Han Y. (2015). SmartPDR: Smartphone-Based Pedestrian Dead Reckoning for Indoor Localization. IEEE Sens. J..

[3] Whittle M.W. (2014). Gait Analysis: An Introduction.

[4] Pham M.H., Elshehabi M., Haertner L., Del Din S., Srulijes K., Heger T., Synofzik M., Hobert M.A., Faber G.S., Hansen C. (2017). Validation of a Step Detection Algorithm during Straight Walking and Turning in Patients with Parkinson’s Disease and Older Adults Using an Inertial Measurement Unit at the Lower Back. Front. Neurol..

[5] Feehan L.M., Geldman J., Sayre E.C., Park C., Ezzat A.M., Yoo J.Y., Hamilton C.B., Li L.C. (2018). Accuracy of Fitbit Devices: Systematic Review and Narrative Syntheses of Quantitative Data. JMIR mHealth uHealth.

[6] Pan D., Dhall A., Liebig A., Stacey B., Shaban H., McDuff D., Yang X., Liu X. (2024). Accuracy of Step Count from Wearable Devices: A Systematic Review and Meta-Analysis. npj Digit. Med..

[7] Vandermeeren S., Steendam H. (2022). Deep-Learning-Based Step Detection and Step Length Estimation With a Handheld IMU. IEEE Sens. J..

[8] Romijnders R., Warmerdam E., Hansen C., Schmidt G., Maetzler W. (2022). A Deep Learning Approach for Gait Event Detection from a Single Shank-Worn IMU: Validation in Healthy and Neurological Cohorts. Sensors.

[9] Foster R.C., Lanningham-Foster L.M., Manohar C., McCrady S.K., Nysse L.J., Kaufman K.R., Padilla D.J., Levine J.A. (2005). Precision and Accuracy of an Ankle-Worn Accelerometer-Based Pedometer in Step Counting and Energy Expenditure. Prev. Med..

[10] Straczkiewicz M., Huang E.J., Onnela J.P. (2023). A “One-Size-Fits-Most” Walking Recognition Method for Smartphones, Smartwatches, and Wearable Accelerometers. npj Digit. Med..

[11] Warden P., Situnayake D. (2019). TinyML: Machine Learning with TensorFlow Lite on Arduino and Ultra-Low-Power Microcontrollers.

[12] Küderle A., Ullrich M., Roth N., Ollenschläger M., Ibrahim A.A., Moradi H., Richer R., Seifer A.K., Zürl M., Sîmpetru R.C. (2024). Gaitmap—An Open Ecosystem for IMU-Based Human Gait Analysis and Algorithm Benchmarking. IEEE Open J. Eng. Med. Biol..

[13] Szczęsna A., Błaszczyszyn M., Amjad A. (2025). Database for Prevalence and Determinants of Frailty and Pre-Frailty in Elderly People with Quantifying Functional Mobility.

[14] Raspberry Pi Ltd. Pico 2W Datasheet: A Microcontroller by Raspberry Pi. Datasheet, 2024. https://pip.raspberrypi.com/documents/RP-008304-DS-1-pico-2-w-datasheet.pdf.

[15] Oudre L., Barrois-Müller R., Moreau T., Truong C., Vienne-Jumeau A., Ricard D., Vayatis N., Vidal P.P. (2018). Template-Based Step Detection with Inertial Measurement Units. Sensors.

[16] Ferri C.P., Jacob K. (2017). Dementia in low-income and middle-income countries: Different realities mandate tailored solutions. PLoS Med..

[17] Tsiakiri A., Plakias S., Giarmatzis G., Tsakni G., Christidi F., Karakitsiou G., Georgousopoulou V., Manomenidis G., Tsiptsios D., Vadikolias K. (2025). Wearable Sensor Technologies and Gait Analysis for Early Detection of Dementia: Trends and Future Directions. Sensors.

[18] Amjad A., Qaiser S., Błaszczyszyn M., Szczęsna A. (2024). The evolution of frailty assessment using inertial measurement sensor-based gait parameter measurements: A detailed analysis. Wiley Interdiscip. Rev. Data Min. Knowl. Discov..

[19] Analog Devices Pedometer Design—3-Axis Digital Accelerometer, AN-1057 Application Note. Application Note AN-1057, 2018. https://www.analog.com/media/en/technical-documentation/application-notes/AN-1057.pdf.

[20] Xu Y., Li G., Li Z., Yu H., Cui J., Wang J., Chen Y. (2022). Smartphone-Based Unconstrained Step Detection Fusing a Variable Sliding Window and an Adaptive Threshold. Remote Sens..

[21] Ma M., Song Q., Gu Y., Li Y., Zhou Z. (2018). An Adaptive Zero Velocity Detection Algorithm Based on Multi-Sensor Fusion for a Pedestrian Navigation System. Sensors.

[22] Cho S.Y., Lee J.H., Park C.G. (2021). A Zero-Velocity Detection Algorithm Robust to Various Gait Types for Pedestrian Inertial Navigation. IEEE Sens. J..

[23] Barth J., Oberndorfer C., Kugler P., Schuldhaus D., Winkler J., Klucken J., Eskofier B. Subsequence dynamic time warping as a method for robust step segmentation using gyroscope signals of daily life activities. Proceedings of the 2013 35th Annual International Conference of the IEEE Engineering in Medicine and Biology Society (EMBC).

[24] Rampp A., Barth J., Schülein S., Gaßmann K.G., Klucken J., Eskofier B.M. (2014). Inertial sensor-based stride parameter calculation from gait sequences in geriatric patients. IEEE Trans. Biomed. Eng..

[25] Figueiredo J., Felix P., Costa L., Moreno J.C., Santos C.P. (2018). Gait event detection in controlled and real-life situations: Repeated measures from healthy subjects. IEEE Trans. Neural Syst. Rehabil. Eng..

[26] Brondin A., Nordström M., Olsson C.M., Salvi D. Open Source Step Counter Algorithm for Wearable Devices. Proceedings of the Companion Proceedings of the 10th International Conference on the Internet of Things (IoT ’20 Companion).

[27] Bosch Sensortec BMA421 Intelligent, Triaxial Acceleration Sensor—Datasheet. Datasheet BST-BMA421-FL000, 2020. https://files.pine64.org/doc/datasheet/pinetime/BST-BMA421-FL000.pdf.

[28] Analog Devices Step Counting Using the ADXL367, AN-2554 Application Note. Application Note AN-2554, 2023. https://www.analog.com/en/resources/app-notes/an-2554.html.

